# Knowledge, Attitudes, and Practices Toward LARS Prevention in Rectal Cancer Patients With Prophylactic Stomas: A Multicenter Cross‐Sectional Study

**DOI:** 10.1002/nop2.70576

**Published:** 2026-05-19

**Authors:** Na Liu, Hong Ying Pi, Yu Ze Sun, Tian Ze Wang

**Affiliations:** ^1^ The First Medical Center of Chinese PLA General Hospital Beijing China; ^2^ Medical Service Training Center of Chinese PLA General Hospital Beijing China; ^3^ PLA Medical College Beijing China

**Keywords:** health education, knowledge, attitudes, and practices (KAP), Low Anterior Resection Syndrome (LARS), mediation analysis, prophylactic stoma

## Abstract

**Aim:**

To assess the current status of knowledge, attitudes, and practices (KAP) related to Low Anterior Resection Syndrome (LARS) prevention among rectal cancer patients with prophylactic stomas.

**Design:**

A multicenter cross‐sectional study was conducted.

**Methods:**

A cross‐sectional survey was administered to 206 patients who underwent prophylactic stoma reversal after sphincter saving rectal cancer surgery at 20 tertiary hospitals in a specific region between January 2024 and January 2025. A validated KAP questionnaire was utilized, comprising three domains: knowledge (12 items), attitudes (10 items), and practices (10 items). Data analysis included descriptive statistics, univariate analysis, multiple linear regression, and mediation analysis.

**Results:**

The total KAP score was 109.39 (20.29) (maximum: 160), with sub‐scores of 38.46 (8.46) (knowledge), 40.02 (4.25) (attitudes), and 30.92 (10.59) (practices). Educational level, residence, and primary caregiver were identified as significant explanatory factors in multiple linear regression analysis of KAP scores (all *p* < 0.05). Attitudes partially mediated the relationship between knowledge and practice (mediation effect: 24.90%, *p* < 0.05). Despite positive attitudes, patients demonstrated moderate knowledge gaps and suboptimal behavioral compliance.

**Patient or Public Contribution:**

Patients contributed data to this study but were not involved in its design, analysis, or reporting.

## 1 Introduction

Low Anterior Resection Syndrome (LARS), characterized by faecal incontinence, urgency, and altered bowel habits, affects 30%~80% of rectal cancer survivors after sphincter sparing surgery (Brock et al. 2023). Prophylactic stomas are commonly used to mitigate anastomotic complications, yet stoma reversal often unmasks LARS symptoms (Sjövall et al. 2023). The LARS score questionnaire can determine the severity of symptoms of LARS, with criteria summarized in Table 1. Five questions have been included, which are incontinence for flatus or liquid stools, frequency of bowel movements, clustering of stools, and urgency. The major LARS can be determined if the score is higher than 30, and minor LARS can be defined if the score is higher than 20 (Emmertsen and Laurberg 2012).

**TABLE 1:** Low anterior resection syndrome score.

Item/symptom	Scoring options & criteria	Score
Incontinence for flatus	Rarely (< 1/week)	0
	Sometimes (1–7/week)	4
	Always (> 7/week, or daily)	7
Incontinence for liquid stool	Rarely (< 1/week)	0
	Sometimes (1–7/week)	3
	Always (> 7/week, or daily)	3
Altered stool frequency	≤ 3 times per day	0
	4–7 times per day	2
	≥ 8 times per day	4
Clustering of stools	No	0
	Yes	2
Urgency	No (able to defer > 15 min)	0
	Yes (unable to defer < 15 min)	6
Total Score & Category	No LARS	0~20
	Minor LARS	21~29
	Major LARS	30~42

Current management strategies (e.g., pelvic floor muscle training (PFMT), dietary modifications) are adherence–dependent, posing challenges for long–term compliance (Kim et al. 2021; Tirelli et al. 2023; van Heinsbergen et al. 2020). The Knowledge‐Attitude‐Practice (KAP) framework is critical for understanding behavioral drivers in chronic disease management (Fenwick et al. 2020; Uriel et al. 2024). This model posits that health‐related behaviors are driven by a sequential process (Zadworna and Stetkiewicz‐Lewandowicz 2023): Knowledge (acquiring information and understanding about a disease) serves as the foundation (Shukla et al. 2021). This knowledge influences Attitude (the beliefs, perceptions, and values one holds regarding the disease and its management), which in turn shapes Practice (the actual adoption of health‐promoting behaviors or adherence to treatment regimens) (Sedlar et al. 2021). In the context of chronic conditions, which require long‐term self‐management, understanding this continuum is essential for developing effective educational and intervention strategies that not only inform patients but also positively influence their attitudes to ultimately foster sustainable behavioural changes. However, evidence on KAP levels and their interplay in LARS prevention, particularly among patients with prophylactic stomas, remains limited. This gap hinders the development of targeted interventions to optimize postoperative care.

## 2 Background

While prophylactic stomas mitigate anastomotic leakage risks, their reversal often unmasks LARS symptoms, creating a critical window for intervention. However, patients' initial focus on stoma care may overshadow awareness of LARS risks, leading to delayed rehabilitation (Annicchiarico et al. 2021; Brock et al. 2023). However, upon reversal, these stomas may lead to symptoms of LARS, presenting significant rehabilitation challenges (Kisielewski et al. 2024). Current management methods, which include PFMT, dietary adjustments, and biofeedback, rely on patient adherence (Brock et al. 2023; Rosen et al. 2019; van der Heijden et al. 2022). However, evidence indicates that this demographic often struggles with knowledge transfer and compliance (Altamimi et al. 2021).

The KAP framework is pivotal for understanding health behavior dynamics in chronic disease management, where knowledge serves as the foundation, attitudes influence motivation, and practices reflect actual health behaviors (Uriel et al. 2024). Despite its utility, limited research has applied the KAP model to LARS prevention, particularly in patients with prophylactic stomas. These patients face unique challenges, as their initial postoperative focus on stoma care may overshadow awareness of LARS risks, leading to delayed intervention post‐reversal (Sjövall et al. 2023). Furthermore, disparities in healthcare access and education, especially in rural or low–literacy populations, exacerbate gaps in preventive practices (Shen et al. 2023). Emerging studies highlight the mediating role of attitudes in bridging knowledge and behavior, emphasizing the need for attitude reinforcement alongside traditional education (Fukuda et al. 2024). However, systematic evaluations of KAP levels, their determinants, and the interplay between psychosocial factors in this context remain scarce (Momaya et al. 2024). This study addresses these gaps by investigating the KAP status of rectal cancer patients with prophylactic stomas, identifying modifiable barriers, and exploring the mediating effect of attitudes. Findings may guide the design of tailored interventions integrating digital tools and caregiver support, potentially reducing LARS related morbidity.

### 2.1 Aim

This study aimed to assess the current status of KAP related to LARS prevention among rectal cancer patients with prophylactic stomas.

### 2.2 Objective

The objective of this research is to conduct a comprehensive analysis of the influencing factors by examining the current status of KAP among patients with prophylactic stomas due to rectal cancer. Specifically, this study endeavours to explore the mediating role that attitude plays in the transformation from knowledge to behavior. Moreover, it is dedicated to delving deeper into the knowledge requirements of this particular patient population. The ultimate goal is to facilitate the more precise implementation of health education initiatives, thereby potentially enhancing the quality of care and the overall well‐being of these patients.

### 2.3 Design

A multicenter cross‐sectional study was conducted, approved by the respective Institutional Review Boards/Ethics Committees of all participating centers.

### 2.4 Participants

Inclusion criteria: (1) age ≥ 18 years; (2) rectal adenocarcinoma diagnosis; (3) prophylactic stoma closure ≥ 1 month post‐surgery. Exclusion criteria: (1) stoma closure failure; (2) non‐LARS related incontinence, such as that caused by enteral nutrition intolerance or severe enteritis; (3) severe postoperative complications, including but not limited to anastomotic leakage requiring reoperation or life‐threatening events like pulmonary embolism. A total of 279 eligible patients were enrolled in this study. Among them, 46 patients were unreachable, and 27 explicitly declined follow‐up. Ultimately, 206 patients were successfully interviewed, yielding a response rate of 73.84%.

### 2.5 Data Collection

Descriptive statistics: Descriptive statistical analysis was performed on the patients' basic information and the scores of each dimension of KAP. Statistical indicators such as the mean, standard deviation, median, and frequency of each variable were calculated to understand the distribution characteristics of the data.

### 2.6 Ethical Considerations

Data were collected through standardized one‐on‐one interviews, and questionnaires were collected on‐site after verification for completeness to ensure the authenticity and effectiveness of the data. This study protocol was approved by the Ethics Committee of Chinese PLA General Hospital (approval number: 574‐01/2023). At the start of the survey, information was given to participants and consent obtained. Data were protected under secure management at the principal investigator's institution. Any data shared were anonymized and accessed through secure password‐protected platforms.

### 2.7 Measurements

This study adopted a questionnaire survey method: (1) General information questionnaire: A self‐designed questionnaire was used to collect socio‐demographic and clinical characteristics. This questionnaire was developed by the research team, comprising a senior enterostomal therapist, physician specialists in colorectal surgery and medical oncology, and a statistician. Its design was informed by a comprehensive review of literature on factors associated with health knowledge, attitudes, and practices in chronic disease and cancer care populations. An initial pool of items was generated based on the review and the specific clinical context of rectal cancer patients with stomas. The items were then discussed and refined through multiple rounds within the research team to ensure content validity, clarity, and relevance to the study aims. The final questionnaire covered key variables including patients' age, gender, educational level, marital status, occupational status, place of residence, living pattern, co‐existing underlying diseases, medical expense payment method, family income, primary caregiver for the stoma, stoma complications, and radiotherapy and chemotherapy status. (2) LARS KAP questionnaire: The LARS KAP questionnaire was adapted from a validated tool for postpartum pelvic floor training (Uriel et al. 2024), through two rounds of Delphi discussions with a panel of 15 experts (4 colorectal surgeons, 9 nursing specialists, 2 rehabilitation therapists). Modifications included rewording items to align with stoma care contexts and adding LARS–specific scenarios. It includes a knowledge dimension (12 items), an attitude dimension (10 items), and a behavior dimension (10 items), with a total of 32 items. The overall Cronbach's α coefficient of the questionnaire was 0.925, and the Cronbach's α coefficients of each dimension were 0.944 for the knowledge dimension, 0.840 for the attitude dimension, and 0.920 for the behavior dimension. The split‐half reliability was 0.894, the test–retest reliability was 0.756, the overall content validity assessment result of the questionnaire was 0.920, and the content validity of each item was 0.933~1.00. The convergent validity and discriminant validity were ideal. The questionnaire used a Likert 5‐level scoring method. All items in the knowledge dimension were scored positively, with scores ranging from 1 (completely disagree) to 5 (completely agree) (Uriel et al. 2024), and a total score ranging from 12 to 60 points. The attitude dimension had both positive and negative scoring items. Positive–scoring items were scored from 1 (completely disagree) to 5 (completely agree), and negative–scoring items were scored from 5 (completely disagree) to 1 (completely agree), with a total score ranging from 10 to 50 points. All items in the behavior dimension were scored positively, with scores ranging from 1 (completely disagree) to 5 (completely agree), and a total score ranging from 10 to 50 points. The total score of the questionnaire ranged from 32 to 160 points. The higher the score, the better the patient's KAP level regarding LARS prevention.

### 2.8 Data Analysis

Data analysis was performed using SPSS 26.0. Descriptive statistics were presented as frequency and percentage for categorical explanatory variables, and mean (standard deviation, SD) for continuous variables. Normality of KAP scores was tested using the Shapiro–Wilk test. Since KAP total scores were non‐normally distributed (*p* < 0.05), non‐parametric tests were used for univariate comparisons: the Mann–Whitney U test for binary explanatory variables and the Kruskal‐Wallis H test for multi‐category explanatory variables. Effect sizes for non‐parametric tests were calculated as eta squared (η^2^).

For multiple linear regression, all multi‐category explanatory variables were entered as dummy/indicator variables with prespecified reference groups. Regression diagnostics included collinearity (variance inflation factor, VIF), residual normality, homoscedasticity, and outlier detection (Cook's distance).

Spearman's correlation was used for bivariate relationships among knowledge, attitude, and practice. Mediation analysis was conducted using the Bootstrap method (5000 resamples) adjusted for educational level, place of residence, and primary caregiver. The indirect effect was significant if the 95% CI did not include zero. Missing data were handled by listwise deletion (missing rate < 5%). Two‐sided *p* < 0.05 was considered statistically significant.

## 3 Results

### 3.1 KAP Status Regarding LARS Prevention

The total KAP score was 109.39 (20.29) (maximum score: 160). The knowledge score was 38.46 (8.46) (score rate: 64.10%), attitude score was 40.02 (4.25) (score rate: 80.04%), and practice score was 30.92 (10.59) (score rate: 61.84%). Patients exhibited positive attitudes but showed moderate knowledge gaps and suboptimal practice compliance (Table 2).

**TABLE 2:** Scores and distribution of each dimension of KAP regarding the LARS.

Dimension	Theoretical range	Mean (SD)	Ranking	Completely disagree *n* (%)	Mostly disagree *n* (%)	Generally agree *n* (%)	Mostly agree *n* (%)	Completely agree *n* (%)
Knowledge (K)	12~60	38.46 (8.46)	Score rate 64.10%					
K1. Have you heard of low anterior resection syndrome of the rectum?	1~5	3.46 (1.17)	15	20 (9.71)	17 (8.25)	55 (26.70)	77 (37.38)	37 (17.96)
K2. Do you know that the clinical manifestations of LARS include urgent defecation, frequent defecation, faecal incontinence, etc.?	1~5	3.32 (1.13)	19	20 (9.71)	20 (9.71)	70 (33.98)	67 (32.52)	29 (14.08)
K3. Do you know the harm caused by LARS?	1~5	2.87 (1.20)	28	35 (16.99)	38 (18.45)	71 (34.47)	42 (20.39)	20 (9.7)
K4. Do you know the mechanism of LARS?	1~5	2.68 (1.22)	29	49 (23.79)	38 (18.45)	63 (30.58)	43 (20.87)	13 (6.31)
K5. Do you know that long – course pelvic radiotherapy before and after surgery is an inducing factor of LARS?	1~5	3.12 (1.03)	23	20 (9.71)	26 (12.62)	80 (38.83)	69 (33.50)	11 (5.34)
K6. Do you know that patients with prophylactic stomas are more likely to develop LARS in terms of aetiology and pathogenesis?	1~5	2.95 (1.12)	27	31 (15.05)	31 (15.05)	71 (34.47)	63 (30.58)	10 (4.85)
K7. Do you know that the occurrence of LARS is related to the dysfunction of the internal anal sphincter?	1~5	3.28 (1.10)	20	17 (8.25)	27 (13.11)	68 (33.01)	69 (33.49)	25 (12.14)
K8. Do you know that pelvic floor muscle training (i.e., Kegel exercise) can prevent the occurrence of LARS?	1~5	3.44 (1.17)	16	18 (8.74)	18 (8.74)	69 (33.49)	57 (27.67)	44 (21.36)
K9. Do you know the method of pelvic floor muscle training (Kegel method, i.e., anal – lifting exercise)?	1~5	3.43 (1.14)	17	18 (8.74)	20 (9.71)	58 (28.15)	75 (36.41)	35 (16.99)
K10. Do you know the benefits and effects of pelvic floor muscle training?	1~5	3.61 (0.86)	10	5 (2.43)	20 (9.71)	73 (35.44)	76 (36.89)	32 (15.53)
K11. Do you know that LARS symptoms can be improved through self – behavior adjustment, diet control, drug treatment, pelvic floor rehabilitation, etc.?	1~5	3.22 (1.18)	22	25 (12.14)	20 (9.71)	76 (36.89)	54 (26.21)	31 (15.05)
K12. Do you know that there are other methods for the prevention and treatment of LARS (such as neuromodulation, colonic irrigation, surgery, etc.)?	1~5	3.08 (1.10)	24	20 (9.71)	23 (11.17)	81 (39.32)	61 (29.62)	15 (7.28)
Attitude and Attitude (A)	10~50	40.02 (4.25)	Score rate 80.04%					
A1. Do you think that involuntary anal exhaust or defecation is a manifestation of LARS?	1~5	4.08 (0.86)	7	1 (0.48)	3 (1.46)	53 (25.73)	70 (33.98)	79 (38.35)
A2. Do you think it is very necessary to master the knowledge related to the prevention of LARS?	1~5	4.25 (0.84)	1	1 (0.48)	6 (2.91)	29 (14.08)	75 (36.41)	95 (46.12)
A3. Do you think it is very necessary to carry out early rehabilitation nursing measures to prevent the occurrence of LARS?	1~5	4.11 (0.96)	6	5 (2.43)	5 (2.43)	40 (19.41)	68 (33.01)	88 (42.72)
A4. Do you think that pelvic floor muscle training can improve the symptoms of LARS?	1~5	3.96 (0.99)	9	5 (2.43)	8 (3.88)	50 (24.27)	70 (33.98)	73 (35.44)
A5. Do you hope to receive health education on pelvic floor muscle training – related knowledge from medical staff?	1~5	4.17 (0.84)	2	1 (0.48)	5 (2.43)	37 (17.96)	78 (37.87)	85 (41.26)
A6. Do you hope to receive continuous guidance on pelvic floor muscle training from medical staff?	1~5	4.12 (0.84)	5	1 (0.48)	5 (2.43)	41 (19.90)	81 (39.32)	78 (37.87)
A7. Do you think you will keep doing pelvic floor muscle training during the stoma reversal preparation stage?	1~5	4.17 (0.81)	3	1 (0.48)	1 (0.48)	43 (20.87)	77 (37.39)	84 (40.78)
A8. Do you think LARS will affect your quality of life?	1~5	4.06 (0.85)	8	1 (0.48)	3 (1.46)	53 (25.73)	74 (35.92)	75 (36.41)
A9. Do you think that if not intervened, the related manifestations of LARS will gradually disappear as the body recovers? (scored 5–1)	5~1	2.97 (1.23)	26	25 (12.14)	52 (25.24)	65 (31.55)	33 (16.02)	31 (15.05)
A10. Do you think that LARS rehabilitation nursing measures are effective measures worthy of long – term adherence?	1~5	4.13 (0.88)	4	1 (0.48)	3 (1.46)	52 (25.24)	63 (30.58)	87 (42.24)
Behavior (P)	10~50	30.92 (10.59)	Score rate 61.84%					
P1. Did you adjust your diet structure after surgery to avoid alcohol, cold and spicy foods, and consume high – fibre and low—fat foods?	1~5	3.53 (1.27)	13	24 (11.65)	16 (7.76)	44 (21.36)	71 (34.47)	51 (24.76)
P2. Did you maintain a positive and optimistic attitude and a good psychological state to cope with post – operative life after surgery?	1~5	3.34 (1.34)	18	34 (16.51)	13 (6.31)	54 (26.21)	59 (28.64)	46 (22.33)
P3. Did you actively learn about the inducing factors, clinical manifestations, and pathogenesis of LARS after surgery?	1~5	3.55 (1.56)	11	45 (21.84)	11 (5.34)	13 (6.31)	60 (29.13)	77 (37.38)
P4. Did you actively learn about the relevant measures for preventing LARS after surgery?	1~5	3.25 (1.51)	21	44 (21.36)	24 (11.65)	35 (16.99)	43 (20.87)	60 (29.13)
P5. Did you actively learn about pelvic floor muscle training – related knowledge after surgery?	1~5	2.44 (1.09)	30	43 (20.87)	75 (36.41)	50 (24.27)	30 (14.57)	8 (3.88)
P6. Can you correctly perform Kegel exercises, straddle – leg anal—lifting exercises, hip—lifting exercises, etc.?	1~5	2.35 (0.99)	32	42 (20.39)	82 (39.80)	52 (25.24)	27 (13.11)	3 (1.46)
P7. Did you keep doing pelvic floor muscle training during the stoma reversal preparation stage?	1~5	3.53 (1.27)	13	24 (11.65)	16 (7.76)	44 (21.36)	71 (34.47)	51 (24.76)
P8. When did you start pelvic floor muscle training after surgery?	1~5	3.55 (1.56)	11	45 (21.84)	11 (5.34)	13 (6.31)	60 (29.13)	77 (37.38)
P9. Did you use zinc oxide or warm – water sitz bath to protect the perianal skin after surgery?	1~5	2.98 (1.37)	25	44 (21.36)	32 (15.53)	46 (22.33)	53 (25.73)	31 (15.05)
P10. Did you take any other intervention measures except pelvic floor muscle training during the stoma reversal preparation stage?	1~5	2.41 (1.45)	31	84 (40.78)	37 (17.96)	27 (13.11)	33 (16.01)	25 (12.14)

### 3.2 Univariate Analysis of Explanatory Variables for KAP Levels

Univariate non‐parametric tests showed that KAP total scores differed significantly by explanatory variables including age group (H = 7.84, *p* = 0.020, *η* ^2^ = 0.060), educational level (H = 10.43, *p* = 0.034, *η* ^2^ = 0.051), occupational status (H = 9.77, *p* = 0.021, *η* ^2^ = 0.058), residence (U = 3714.51, *p* = 0.004, *η* ^2^ = 0.020), primary caregiver (H = 15.92, *p* < 0.001, *η* ^2^ = 0.078), stoma complications (U = 2561.06, *p* = 0.019, *η* ^2^ = 0.012), stoma duration (H = 18.66, *p* < 0.001, *η* ^2^ = 0.091), and comorbidities (H = 8.41, *p* = 0.015, *η* ^2^ = 0.061). No significant differences were found for gender, marital status, monthly family income, or radiotherapy/chemotherapy status (all *p* > 0.05). Detailed results are shown in Table 3.

**TABLE 3:** Univariate analysis of KAP total scores by explanatory variables (*N* = 206).

Explanatory variables	Categories	*N*	KAP total score, mean (SD)	Test statistic	*p*	Effect size (η^2^)
Gender	Male	146	109.49 (20.32)	*U* = 4372.03	0.937	< 0.001
	Female	60	109.18 (20.37)			
Age	18–59	99	107.65 (18.95)	*H* = 7.84	0.020	0.060
	60–79	97	113.17 (18.51)			
	≥ 80	10	99.00 (22.99)			
Educational level	Primary school	38	109.61 (20.48)	*H* = 10.43	0.034	0.051
	Junior high	75	105.99 (19.28)			
	High/technical	37	113.38 (20.94)			
	Junior college	25	102.96 (23.26)			
	University and above	31	117.84 (16.20)			
Marital status	Married	198	109.47 (19.99)	*U* = 776.5	0.813	0.001
	Unmarried/Divorced/Widowed	8	107.25 (16.77)			
Occupational status	Employed	44	102.11 (17.59)	*H* = 9.77	0.021	0.058
	Retired	119	111.38 (20.04)			
	Unemployed	27	107.07 (21.28)			
	Others	16	118.63 (22.46)			
Residence	Urban	143	112.18 (20.36)	*U* = 3714.51	0.004	0.020
	Rural	63	103.10 (18.79)			
Primary caregiver	Self‐care	17	93.47 (13.36)	*H* = 15.92	< 0.001	0.078
	Spouse	100	109.03 (20.20)			
	Children	81	112.26 (20.74)			
	Others	8	118.88 (11.99)			
Monthly income	< 5000	43	103.44 (20.60)	*H* = 4.59	0.100	0.010
	5000–10,000	128	111.02 (20.28)			
	> 10,000	35	110.77 (19.23)			
Stoma complications	None	36	102.11 (17.66)	*U* = 2561.06	0.019	0.012
	Present	170	110.94 (20.52)			
Radiotherapy/Chemo	None	72	108.25 (20.19)	*H* = 6.14	0.106	0.013
	RT only	12	101.25 (17.67)			
	CT only	98	109.16 (21.42)			
	Both	24	117.88 (14.52)			
Stoma duration	≤ 3 m	41	104.78 (24.14)	*H* = 18.66	< 0.001	0.091
	3–6 m	94	115.49 (17.70)			
	6–12 m	62	105.24 (19.41)			
	> 12 m	9	95.44 (14.81)			
Comorbidities	None	86	106.45 (20.42)	*H* = 8.41	0.015	0.061
	One	86	114.10 (17.16)			
	Two+	34	104.94 (24.97)			

### 3.3 Multiple Linear Regression Analysis for Explanatory Factors

Multiple linear regression analysis was performed to identify factors associated with KAP total scores. The overall regression model was statistically significant (*F*(19, 186) = 4.271, *p* < 0.001), explaining 28.7% of the variance in KAP scores (*R* ^2^ = 0.287). Dummy coding was applied to all multi‐category explanatory variables (Table 4). After adjusting for all covariates, educational level, place of residence, and primary caregiver remained significant explanatory factors (all *p* < 0.05; Table 5). Regression diagnostics confirmed no multicollinearity (all VIF < 2.0), approximate normality of residuals, homoscedasticity, and no influential outliers (all Cook's distance < 1). Robust standard errors confirmed stable results (Table 5).

**TABLE 4:** Dummy variable coding of explanatory variables for KAP analysis among rectal cancer patients with prophylactic stomas for LARS prevention.

Variable	Category	Dummy variable code	Reference group
Age	18–59 years	(Reference)	18–59 years
	60–79 years	Age1 (1 = yes, 0 = no)	
	≥ 80 years	Age2 (1 = yes, 0 = no)	
Educational level	Primary school	Edu1 (1 = yes, 0 = no)	University and above
	Junior high school	Edu2 (1 = yes, 0 = no)	
	High school/technical secondary school	Edu3 (1 = yes, 0 = no)	
	Junior college	Edu4 (1 = yes, 0 = no)	
	University and above	(Reference)	
Occupational status	Employed	(Reference)	Employed
	Retired	Occ1 (1 = yes, 0 = no)	
	Unemployed	Occ2 (1 = yes, 0 = no)	
	Others	Occ3 (1 = yes, 0 = no)	
Place of residence	Urban	Res1 (1 = yes, 0 = no)	Rural
	Rural	(Reference)	
Primary caregiver	Self‐care	(Reference)	Self‐care
	Spouse	Care1 (1 = yes, 0 = no)	
	Children	Care2 (1 = yes, 0 = no)	
	Others	Care3 (1 = yes, 0 = no)	
Stoma complications	No	(Reference)	No
	Yes	Comp1 (1 = yes, 0 = no)	
Duration of stoma‐wearing	≤ 3 months	(Reference)	≤ 3 months
	> 3–≤ 6 months	Dur1 (1 = yes, 0 = no)	
	> 6–≤ 12 months	Dur2 (1 = yes, 0 = no)	
	> 12 months	Dur3 (1 = yes, 0 = no)	
Presence of underlying diseases	None	(Reference)	None
	One	Comorb1 (1 = yes, 0 = no)	
	Two or more	Comorb2 (1 = yes, 0 = no)	

**TABLE 5:** Multivariate linear regression analysis of explanatory variables influencing KAP levels.

Variable	B	SE	β	*t*	*p*	VIF
(Constant)	111.245	22.817	—	4.875	< 0.001	—
Age1 (60–79 vs. 18–59)	5.517	3.124	0.118	1.766	0.079	1.29
Age2 (≥ 80 vs. 18–59)	−8.649	7.216	−0.081	−1.199	0.232	1.27
Edu1 (Primary vs. University+)	−9.241	2.104	−0.214	−4.392	< 0.001	1.66
Edu2 (Junior high vs. University+)	−8.573	2.018	−0.198	−4.248	< 0.001	1.62
Edu3 (High/technical vs. University+)	−5.826	1.975	−0.134	−2.950	0.003	1.58
Edu4 (Junior college vs. University+)	−7.914	2.082	−0.181	−3.799	< 0.001	1.64
Occ1 (Retired vs. Employed)	2.047	3.862	0.048	0.530	0.596	1.44
Occ2 (Unemployed vs. Employed)	−1.135	4.107	−0.025	−0.276	0.783	1.41
Occ3 (Others vs. Employed)	4.219	4.526	0.082	0.932	0.352	1.37
Res1 (Urban vs. Rural)	10.652	4.713	0.243	2.260	0.025	1.15
Care1 (Spouse vs. Self‐care)	7.245	2.614	0.247	2.771	0.006	1.59
Care2 (Children vs. Self‐care)	9.017	2.683	0.296	3.361	0.001	1.63
Care3 (Others vs. Self‐care)	9.432	3.015	0.302	3.128	0.002	1.56
Comp1 (Yes vs. No)	6.984	6.827	0.132	1.023	0.307	1.19
Dur1 (> 3–≤ 6 vs. ≤ 3)	1.572	5.106	0.036	0.308	0.758	1.68
Dur2 (> 6–≤ 12 vs. ≤ 3)	−0.841	4.983	−0.019	−0.169	0.866	1.65
Dur3 (> 12 vs. ≤ 3)	−1.964	5.628	−0.042	−0.349	0.727	1.61
Comorb1 (One vs. None)	3.215	4.862	0.114	0.661	0.509	1.43
Comorb2 (Two or more vs. None)	−2.846	5.417	−0.096	−0.525	0.600	1.44
Model Summary	*F* = 4.271, *p* < 0.001					
	*R* ^2^ = 0.287, Adjusted					
	*R* ^2^ = 0.240					

*Note:* Dependent variable: KAP total score; *n* = 206; VIF < 2.0 for all variables, indicating no multicollinearity.

### 3.4 Correlation and Mediation Analyses

Spearman's correlation showed positive pairwise correlations among knowledge, attitude, and practice scores (all *p* < 0.01; Table 6).

**TABLE 6:** Correlation Analysis of Domain–Specific Scores for Knowledge, Attitudes, and Practice in LARS prevention.

Variable	Knowledge	Attitude	Practice
Knowledge	1		
Attitude	0.422**	1	
Practice	0.433**	0.371**	1

** *p* < 0.01.

**FIGURE 1:**
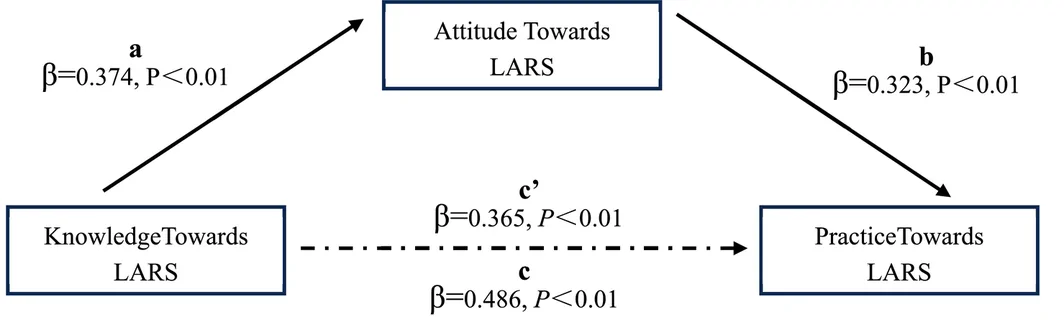
Mediation model of knowledge, attitude, and practice regarding LARS prevention (*N* = 206), adjusted for educational level, place of residence, and primary caregiver.

Bootstrap mediation analysis (5000 resamples) confirmed that attitude played a partial mediating role between knowledge and practice, with an indirect effect of 0.121 (95% CI: 0.034~0.172, *p* < 0.001), accounting for 24.90% of the total effect. The mediation model adjusted for education level, place of residence, and primary caregiver (Table 7; Figure 1).

**TABLE 7:** Mediating effect analysis of the attitude dimension between the knowledge and practice dimensions.

Item	Symbol	Meaning	Effect value	95% CI		Standard error SE value	*z*/*t*	*p*	Conclusion	Effect proportion
				Lower	Limit					
K→A→P	a*b	Indirect Effect	0.121	0.034	0.172	0.036	3.361	< 0.001	Partial mediation	24.90%
K→A	a	X→M	0.374	0.278	0.470	0.048	7.792	< 0.001		
A→P	b	M→Y	0.323	0.112	0.534	0.108	2.982	0.003		
K→P	c’	Direct Effect	0.365	0.190	0.540	0.089	4.101	< 0.001		
K→P	c	Total Effect	0.486	0.328	0.644	0.080	6.075	< 0.001		

*Note:* The mediation model was adjusted for educational level, place of residence, and primary caregiver.

### 3.5 Knowledge Needs Regarding LARS Prevention

The top three factors affecting rehabilitation behaviors (mainly PFMT) were: time consumption and lack of patience and perseverance (55.34%), insufficient understanding of the importance of pelvic floor exercises and poor compliance (33.98%), and unclear exercise methods or uncertainty about correct execution (27.19%). The top three sources of LARS prevention knowledge were: oral education by doctors and nurses (85.44%), postoperative telephone follow–up (33.50%), and printed materials (30.10%). The preferred methods for obtaining LARS prevention knowledge were: video and animation (84.47%), written materials and brochures (50.97%), and WeChat public account or group messages (49.51%). The top three types of relevant knowledge that patients most desired to obtain during the stoma reversal preparation stage were: the methods of pelvic floor muscle training (90.29%), dietary management (84.95%), and the content of observation and nursing for the dynamic changes of the stoma (46.60%), as detailed in Table 8.

**TABLE 8:** Sources of information and educational needs for LARS prevention in rectal cancer patients with prophylactic stomas.

Items	*n*	Proportion (%)
Top three factors affecting rehabilitation behavior		
Time consumption, lack of patience and perseverance	114	55.34
Insufficient understanding of the importance of pelvic floor exercise and poor compliance	70	33.98
Unawareness of exercise methods or uncertainty about the correctness of movements	56	27.18
Top three sources of knowledge for preventing LARS		
Oral education by doctors and nurses	176	85.44
Post – operative telephone follow – up	69	33.50
Paper – based promotional materials	62	30.10
Top three preferred ways to obtain knowledge related to preventing LARS		
Promotional videos and animated videos	174	84.47
Written materials and brochures	105	50.97
Text messages or notifications via social media platforms or mobile messaging applications	102	49.51
Top three contents of knowledge expected to be obtained during the stoma reversal preparation stage		
Pelvic floor muscle training methods	186	90.29
Diet management	175	84.95
Observation and care of dynamic changes of stoma	103	46.60

## 4 Discussion

### 4.1 Current Status of KAP Levels in LARS Prevention

This study revealed suboptimal overall KAP levels among rectal cancer patients with prophylactic stomas, with significant disparities across dimensions (knowledge: 64.10%; attitude: 80.04%; practice: 61.84%). These findings suggest that despite patients' positive attitudes toward rehabilitation, substantial gaps persist in their cognitive preparedness and practical capacity for LARS prevention (Brock et al. 2023). Unlike studies in centralized healthcare systems, the findings highlight systemic gaps in the post‐stoma care within the study setting, particularly in knowledge dissemination—a challenge less emphasized in high‐resource settings. While continuity of care challenges are documented globally (Su et al. 2021), the data uniquely attribute knowledge gaps to fragmented post‐discharge support systems in this context (Hu, Zhang, et al. 2024; Wang et al. 2018), where standardized LARS education protocols are absent. This presents a critical target for policy reform in similar healthcare environments facing such fragmentation. Notably, the lowest scores in the knowledge dimension underscore the urgent need for systematic education on LARS prevention in clinical practice. The marginally lower practice scores compared to knowledge scores reflect challenges in translating awareness into action, potentially due to postoperative physical limitations or insufficient social support (Kazim et al. 2021). Knowledge gaps may directly hinder adherence to critical interventions, while the disconnection between attitude and practice highlights inefficiencies in the “knowledge to action” conversion mechanism (Kazim et al. 2021). Consequently, a structured health education framework is imperative to bridge the gap between patients' rehabilitation expectations and their actual capabilities through enhanced knowledge dissemination and behavioral guidance.

### 4.2 Impact of Population Heterogeneity on KAP Levels

The demographic profile of our cohort reflects the typical epidemiology and social context of patients undergoing rectal cancer surgery with prophylactic stomas in the studied population. Our sample exhibited overrepresentations of males, married individuals, and urban residents. First, the male predominance aligns with the known higher incidence of rectal cancer among males (Huang et al. 2022). Second, the high marriage rate is directly linked to the disease's typical age of onset (cohort median age: 60.0 years), which mirrors the marital pattern of that age group in the general population. Third, the urban overrepresentation is likely attributable to the location of the participating tertiary hospitals in urban centers. While this sample accurately represents a key patient group seeking specialized care, these demographic characteristics also directly influenced the observed KAP patterns. Specifically, Education level, residence, and primary caregiver type significantly influenced KAP outcomes. In contrast, rural patients exhibited lower KAP levels (Alhowaymel et al. 2023), potentially attributable to uneven healthcare resource distribution and limited health education coverage (Fu et al. 2024). The interplay between education level and residence warrants attention (Jackson and Haile 2023). Residence may influence KAP not only directly but also indirectly by shaping educational opportunities and health awareness, with education level serving as a potential mediator. This speculation underscores the complex socio‐ecological context of patient education (Zhou and Li 2021). Future longitudinal studies are needed to disentangle the direct and indirect effects of socioeconomic and geographic factors, thereby clarifying the mediating mechanisms to better inform targeted interventions (Goldman et al. 2022). Additionally, variations in caregiver roles (e.g., family members vs. professional caregivers) indirectly affected behavioral motivation through differences in social support systems. Current guidelines lack rural specific strategies. Our proposed community based follow ups address this gap by leveraging local healthcare networks (Fu et al. 2024). This includes developing simplified, culturally tailored educational materials for low–literacy and rural populations, with the addition of community based follow ups. Simultaneously, professional training for caregivers should be carried out to enhance their role as “health behavior facilitators”.

### 4.3 Mediating Role of Attitude in Knowledge‐To‐Practice Transition

Significant positive correlations were observed among knowledge, attitude, and practice. Mediation analysis confirmed that attitude partially mediated the relationship between knowledge and practice (mediation effect: 24.90%), aligning with the classic KAP theoretical framework and health behavior process models (Hiew and Low 2024). Even after adjusting for covariates such as education, attitude retained its mediating role, further supporting its independence in promoting health behaviors (Zhang et al. 2024). Specifically, patients' attitudes about LARS prevention were shaped not only by knowledge but also by psychological traits and socioenvironmental factors (Cho et al. 2021). For instance, some patients discontinued PFMT despite recognizing its importance, citing concerns about efficacy or lack of family support (Hu, Qin, et al. 2024). These findings necessitate a paradigm shift in clinical education – from passive knowledge delivery to active attitude reinforcement through motivational interviewing and cognitive – behavioral techniques – to foster intrinsic motivation and sustainable health practices (Elster et al. 2021). Our mediation analysis not only validates the KAP framework but extends it by identifying attitude as an independent driver of practice beyond knowledge, a finding critical for designing attitude centric interventions.

### 4.4 Patient Educational Needs and Optimisation of Health Education Pathways

Patients prioritized knowledge on PFMT methods (90.29%) and dietary management (84.95%), reflecting a demand for proactive postoperative interventions. While PFMT is a cornerstone of LARS prevention, its efficacy depends on adherence and procedural accuracy (Hong et al. 2025). Key barriers included time constraints (55.34%), insufficient awareness of exercise importance (33.98%), and unclear execution guidelines (27.19%), consistent with findings by Báez‐Díaz and Crisóstomo (2023). This underscores the insufficiency of knowledge‐based interventions alone; psychological support and personalized guidance are critical (Chen et al. 2024). Notably, although traditional verbal counselling by healthcare providers remained the primary information source (85.44%), patients preferred video–based materials (84.47%) and written guides (50.97%), exposing limitations of unidirectional communication in conventional education (Chen et al. 2024). Multimedia tools such as instructional animations demonstrating standardized PFMT techniques can enhance knowledge retention, reduce ineffective practices, and provide flexible learning with real–time feedback (Antônio et al. 2023). To optimize health education, a comprehensive strategy should be implemented, including the development of modular programs that cover PFMT, dietary management, and stoma care to address specific patient knowledge gaps, the utilization of diverse media such as videos and mobile apps to accommodate varying learning preferences, and the integration of telehealth platforms for dynamic follow ups (Phillips et al. 2024). Additionally, support systems should be established to implement goal setting and incentive mechanisms, thereby mitigating adherence barriers related to time constraints and motivation. To our knowledge, this is the first study to quantify the preference hierarchy for educational tools (video > written > verbal) in stoma patients, providing actionable evidence for multimedia‐based intervention design.

### 4.5 Study Limitations

This study has several limitations. First, it was a cross‐sectional design, which precludes the establishment of causality. Second, we did not assess standardized clinical performance status measures, such as the ASA physical status classification or WHO performance scale, which could provide a more nuanced characterization of the cohort's overall health. Future prospective studies would benefit from incorporating these validated scales to better control for and understand the influence of functional status on KAP outcomes.

## 5 Conclusion

This study systematically revealed the current status and influencing factors of KAP regarding LARS prevention in patients with preventive stoma for rectal cancer, emphasizing the key role of attitudes in the knowledge to practice transformation. Clinical practice should integrate multi‐level intervention strategies, including knowledge enhancement, attitude reshaping, and behavioral support, while leveraging digital tools to innovate education models. Future research should further explore the long‐term effects of KAP interventions on LARS incidence and quality of life, providing evidence‐based support for precision nursing.

## Author Contributions

Na Liu, Yu Ze Sun, Tian Ze Wang made substantial contributions to conception and design, or acquisition of data, or analysis and interpretation of data. Na Liu involved in drafting the manuscript or revising it critically for important intellectual content. Na Liu, Hong Ying Pi, Yu Ze Sun, Tian Ze Wang given final approval of the version to be published. Each author should have participated sufficiently in the work to take public responsibility for appropriate portions of the content. Hong Ying Pi, Na Liu agreed to be accountable for all aspects of the work in ensuring that questions related to the accuracy or integrity of any part of the work are appropriately investigated and resolved.

## Funding

This work was supported by the Youth Independent Innovation Science Fund – Growth Project of Chinese PLA General Hospital (Grant 22QNCZ012).

## Ethics Statement

This study protocol was approved by the Ethics Committee of Chinese PLA General Hospital (approval number: 574‐01/2023). All participants provided written informed consent prior to enrollment.

## Conflicts of Interest

The authors declare no conflicts of interest.

## Data Availability

The data that support the findings of this study are partly available on request to the corresponding author. The data are not publicly available due to privacy or ethical restrictions.
